# The Hidden Enemy: A Case Report of Early-Onset Rheumatoid Vasculitis Masquerading as Peripheral Artery Disease

**DOI:** 10.31138/mjr.230625.erv

**Published:** 2026-03-01

**Authors:** Shrreya Agarawal, Gorantla Sai Susmitha, Joydeep Samanta, Jhasaketan Meher, Vinay R. Pandit

**Affiliations:** Department of General Medicine, All India Institute of Medical Sciences, Raipur, Chhattisgarh, India

**Keywords:** rheumatoid arthritis, vasculitis, peripheral vascular disease, atherosclerosis

## Abstract

Rheumatoid vasculitis (RV) is a rare, severe extra-articular manifestation of rheumatoid arthritis (RA), typically occurring after long-standing erosive disease. Advances in RA therapy have reduced its incidence; however, RV can occasionally present early in high-risk patients. We report a man in his thirties from Central India with a 3-year history of inadequately treated seropositive RA, who presented with progressive polyarthritis, neuropathy, and distal ischemic changes. Over five months, he developed worsening joint pain and swelling, followed by tingling, numbness, and subsequently dry gangrene of the 2^nd^–5^th^ toes of the left foot. Examination revealed palpable peripheral pulses, normal ankle–brachial index, active synovitis in 24/28 joints, and sensorimotor deficits with weakness and absent ankle reflex. Laboratory evaluation showed anaemia, thrombocytosis, markedly elevated ESR and CRP, and high RF (288 U/mL) and ACPA (>300 U/mL). ANA and ANCA were negative. CT angiogram ruled out peripheral arterial occlusion, and echocardiography excluded cardiac emboli. Nerve conduction study demonstrated sensorimotor axonal neuropathy. Sural nerve biopsy confirmed vasculitis with axonal damage and perivascular inflammation. A diagnosis of rheumatoid vasculitis was made. Treatment with high-dose intravenous methylprednisolone, followed by oral prednisolone, rituximab, and methotrexate, led to rapid control of joint inflammation and halting of gangrene progression. At 6-month follow-up, the patient was asymptomatic on methotrexate 25 mg/week with steroids tapered off, and no further vascular events. This case highlights that RV may rarely occur early in seropositive RA with poorly controlled disease. Prompt recognition, biopsy confirmation, and aggressive immunosuppression are crucial for favourable outcomes.

## INTRODUCTION

Rheumatoid arthritis (RA) is a chronic autoimmune disease, causing joint inflammation, primarily in the hands and feet. Rheumatoid factor (RF) and anti-citrullinated protein antibodies (ACPA) are linked to more severe disease, with extra-articular manifestations affecting the skin, vessels, nerves, eyes, lungs, and other organs.^1^ Rheumatoid vasculitis (RV) is a rare but serious complication involving blood vessel inflammation, leading to skin ulcers and neuropathy. Advances in treatment have significantly reduced RV incidence, improving outcomes and reducing mortality in RA patients.

## CASE PRESENTATION

**Figure 1.**
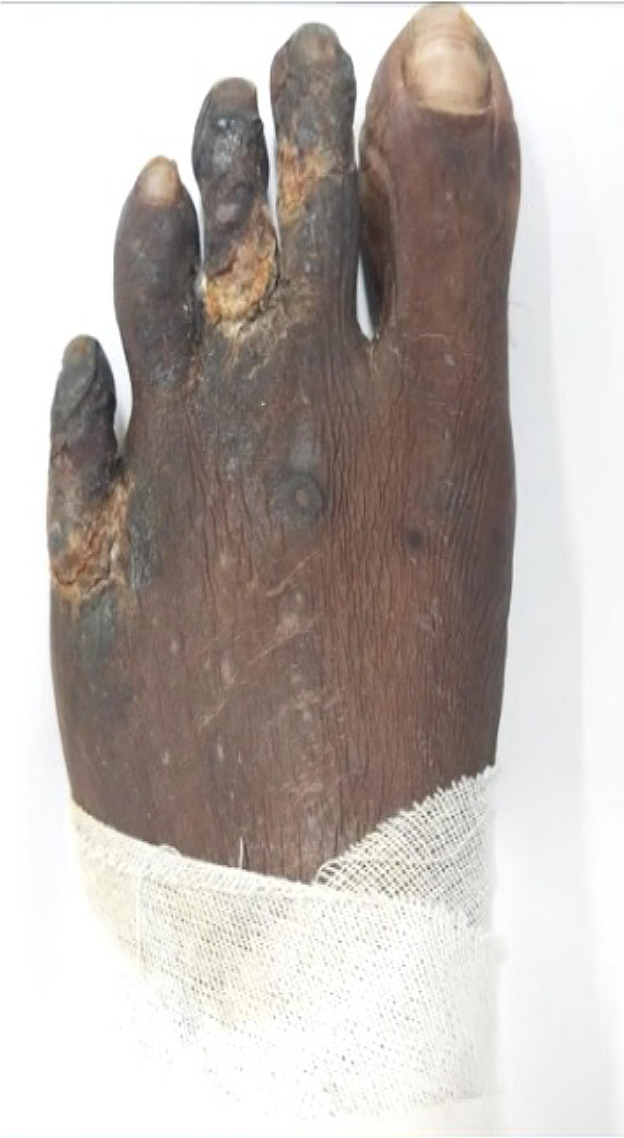
Joint pain and swelling, along with a two-month history of tingling and numbness over the toes of his left foot, which culminated in blackish discolouration (dry gangrene) of the 2nd, 3rd, 4th, and 5th toes over the past month.

A man in his thirties from Central India, diagnosed with rheumatoid arthritis (RA) three years prior, presented with progressive joint symptoms and peripheral neuropathy. His history revealed infrequent use of methotrexate and no other significant comorbidities such as diabetes, smoking, or peripheral vascular disease. He reported five months of worsening joint pain and swelling, along with a two-month history of tingling and numbness over the toes of his left foot, which culminated in blackish discolouration (dry gangrene) of the 2nd, 3rd, 4th, and 5th toes over the past month (**Figure 1**). Importantly, there were no systemic features such as fever, rash, or preceding episodes of Raynaud’s phenomenon.

## INVESTIGATIONS

On examination, he was hemodynamically stable, with palpable peripheral pulses and a normal ankle-brachial index (ABI). His left foot showed dry gangrene of multiple toes, accompanied by non-healing dorsal ulcers, a limited range of motion, and sensory loss over the affected area. Joint examination revealed active synovitis (24 out of 28 joints tender/swollen) without deformities. Neurological examination of the left leg demonstrated motor weakness (3/5 strength in ankle dorsiflexors and plantar flexors), absent ankle reflex, and loss of multiple sensory modalities up to the ankle. Laboratory investigations showed a normocytic normochromic anaemia (Hb 7.14 g/dL), marked thrombocytosis (platelet count 9.41 lakh/mm^3^), elevated inflammatory markers (ESR 80 mm/hr, CRP 104 mg/L), and high titres of rheumatoid factor (RF 288 U/mL) and anti-citrullinated protein antibody (ACPA >300 U/ mL). Other autoimmune markers (ANA, anti-PR3/MPO, serum cryoglobulin) were negative. All investigation reports are enumerated in **Table 1**.

**Table 1.** Blood investigation reports.

**Parameters (Units of measurement**)	**Value**	**Normal range**
Haemoglobin (gm/dL)	7.14	12.0–16.0
Total leukocyte count (× 10^9^/L )	8.6	4.0–11.0
Platelet count (× 10^9^/L )	941	150–450
Erythrocyte sedimentation rate (mm/1^st^ hour )	80	2–20
C reactive protein (CRP) (mg/L)	104	<6
Packed Cell Volume/Haematocrit (%)	21.6	(36–46)
Mean Corpuscular Volume (fl)	93.6	80–100
Mean Corpuscular Haemoglobin (pg)	30.9	26–34
Mean Corpuscular Haemoglobin Concentration (gm/dL)	33.5	31–37
Serum bilirubin (mg/dL)	1.2	0.2–1.2
SGPT (U/L)	46	2–41
SGOT (U/L)	38	2–40
ALP (U/L)	126	40–128
Total protein (gm/dL)	6.3	6.0–8.3
Albumin (gm/dL)	3.0	3.4–4.8
Serum urea (mg/dL)	22.8	10–50
Serum creatinine (mg/dL)	0.86	0.5–1.2
ANA by IIF	Negative	Negative
RF (Units/mL)	288	<20
Anti-CCP (Units/mL)	>300	<12
Anti-PR3 by ELISA (Units/mL)	3.6	<20
Anti-MPO by ELISA (Units/mL)	2.286	<20
Serum Cryoglobulin	Not detected	-
HBsAg (qualitative) by ELISA	Negative	Negative
Anti-HCV (qualitative) by ELISA	Negative	Negative

SGPT: Serum glutamic-pyruvic transaminase; SGOT: Serum glutamic-oxaloacetic transaminase; ALP: Alkaline phosphatase; ANA: Anti-nuclear antibody; IIF: indirect immunofluorescence; RF: Rheumatoid factor; Anti CCP: Anti cyclic citrullinated peptide; ELISA: Enzyme-Linked Immunosorbent Assay; PR3: Proteinase 3; MPO: Myeloperoxidase; HBsAg: Hepatitis B surface antigen; anti HCV: Anti Hepatitis C antibody.

## DIFFERENTIAL DIAGNOSIS

**Figure 2.**
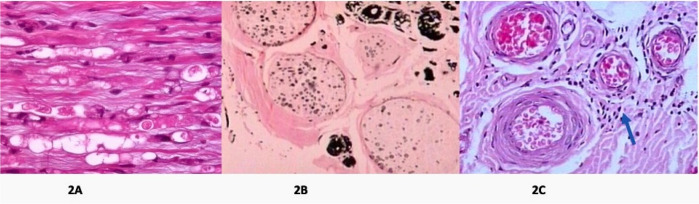
Sural nerve biopsy that showed acute axonal damage, myelinated fibre loss, and perivascular inflammation.

Considering distal asymmetric dry gangrene in a middle-aged patient and the presence of neuropathy, peripheral vascular disease, systemic vasculitides (secondary vasculitis like rheumatoid vasculitis or primary systemic vasculitis), or embolic occlusion of lower limb arteries were kept as possible differentials. However, the presence of peripheral pulses, normal ABI and neuropathy was odd for peripheral vascular diseases. On the other hand, the short duration of RA was odd for rheumatoid vasculitis, which is generally a late complication of RA. Because of toe gangrene, a CT angiogram of bilateral lower limbs was done, which ruled out any thrombotic causes or peripheral arterial disease. Echocardiography was done to rule out any vegetations or cardiac emboli, and it also came out to be normal. Considering the clinical findings suggestive of peripheral neuropathy, a nerve conduction study was performed, which showed an absent compound muscle action potential (CMAP) in both bilateral peroneal and tibial nerves, and absent sensory nerve action potential (SNAP) responses in both bilateral sural nerves, consistent with sensorimotor axonal neuropathy. Keeping a possibility of vasculitic neuropathy, a sural nerve biopsy was done, which showed acute axonal damage, myelinated fibre loss, and perivascular inflammation, thereby confirming the diagnosis of vasculitis (**Figure 2**).

Considering vasculitic neuropathy and toe gangrene in a background of inadequately treated seropositive RA, a diagnosis of secondary vasculitis due to RA (rheumatoid vasculitis) was made. Though rheumatoid vasculitis typically occurs in patients with long-standing RA, it can occasionally present earlier, especially in patients with high RF and ACPA titres and poorly controlled disease.

## TREATMENT

Management was initiated urgently due to the severity of vasculitic involvement. High-dose intravenous methylprednisolone (750 mg daily for 3 days) was given, followed by oral prednisolone (1 mg/kg/day) with gradual tapering. Rituximab (two doses of 1 g two weeks apart) was introduced as a steroid-sparing agent. Methotrexate was continued at a weekly dose of 15 mg, and appropriate analgesia was provided for pain control.

### Outcome and Follow-up

With the introduction of steroids and Rituximab, there was no further progression of gangrene. Also, joint symptoms subsided gradually. The patient was discharged on oral Methotrexate. The steroid was steadily tapered, and the Methotrexate dose was escalated. After six months of follow-up, she was doing well on Methotrexate 25 mg/week without any joint symptoms, and there was no further progression of gangrene.

## DISCUSSION

Rheumatoid vasculitis (RV) is a rare but severe extra-articular manifestation of rheumatoid arthritis (RA), with a 30-year incidence of approximately 3.6%.^2^ It typically arises after a decade or more of RA, often when joint disease is less active.^3^ However, exceptions occur, as seen in this patient from Central India, who developed RV early in the disease course with high joint activity and no erosions, a presentation that has been documented in the literature.^4^ The underlying mechanism of RV involves the deposition of circulating immune complexes, particularly IgG rheumatoid factor (RF) and other autoantibodies, into the walls of small- and medium-sized vessels. This immune complex deposition activates the complement system, leading to inflammation, endothelial injury, and damage to the vessel wall. This pathophysiology aligns with the observation that RV patients often have very high RF titres and low complement levels compared to those with active synovitis alone.^5^ Risk factors for RV include long-standing erosive RA, high seropositivity (RF and ACPA), male sex, smoking, and certain genetic predispositions linked to HLA class I and II alleles.^6^ The current patient, with high RF and ACPA levels and male gender, fits into this high-risk category despite his relatively short RA duration. Clinically, RV presents with a wide range of manifestations. Cutaneous signs (65–88%), neurological involvement (35–63%), and cardiac manifestations (33%) are the most common.^7^ Less commonly, the cerebral, hepatic, gastrointestinal, and large vessels may also be involved, leading to significant morbidity and mortality.^8–12^ In this case, the patient presented with dry gangrene of the toes and sensorimotor peripheral neuropathy, without systemic features such as fever or rash. Diagnosis relies on clinical assessment, supportive serology (high RF, ACPA, and inflammatory markers), and confirmatory tissue biopsy. In this patient, sural nerve biopsy demonstrated perivascular inflammation and axonal damage, confirming vasculitic neuropathy. The Scott and Bacon criteria support the diagnosis in patients with RA who develop features such as gangrene, mononeuritis multiplex, or necrotising arteritis without alternative explanations.^13^ Biopsy remains an important tool in the diagnosis of RV- histopathology of gangrenous tissue or, sural nerve biopsy in case of neuropathy can help in this context.

Management depends on disease severity. Limited cutaneous disease can be treated with oral corticosteroids and DMARDs like methotrexate. However, severe or organ-threatening disease, as in this case, requires high-dose corticosteroids and additional immunosuppressants like rituximab or cyclophosphamide.^13,14^ Despite therapy, RV remains associated with a high risk of mortality, with approximately 40% of patients dying within five years due to infections or organ failure.^15^

## CONCLUSION

Although rheumatoid vasculitis is a late complication of erosive RA, it can present at any time during the disease process. The development of non-healing ulcers, digital gangrene, and peripheral nervous system involvement should raise suspicion of RV. Sometimes it can mimic the presentation of peripheral vascular disease. Accurate diagnosis of rheumatoid vasculitis requires careful consideration and exclusion of conditions that mimic vasculitis, with biopsy as the definitive diagnostic tool. Early diagnosis and treatment with aggressive immunomodulation are imperative for positive outcomes.
